# Supplementary material for: YAP1 depletion enhances TAZ and its complexation with TEAD4 and AP-1 heterodimer C-JUN/FOSB in gastric cancer progression and metastasis
Source: Cancer Heterog Plast. Author manuscript; Available in PMC 2026 Jul 7. (PMC13334769; doi:10.47248/chp2603010003)
Supplement: Supplementary Material — Figure S1. YAP1 and TAZ are highly expressed and significantly correlated in GC tumor tissues and PDXs. Figure S2. Co-expression of YAP1 and TAZ in representative GCPM samples. Figure S3. High expression of TEAD1-TEAD4, the transcription factors (TFs) for YAP1/TAZ, in primary tumor and metastatic tissues. Figure S4. Effects of YAP1 ASO on YAP1 targets and on tumor cell colony formation. Figure S5. TAZ ASO specifically suppress TAZ expression and inhibit GA0518 cell colony formation. Figure S6. Cotreatment GC tumor cells using YAP1, and TAZ ASOs effectively suppress both YAP1 and TAZ expression and suppress tumor cell growth in YAP1-high GC cells. Figure S7. ASO co-targeting of YAP and TAZ significantly attenuated tumor growth in an additional PDX. Figure S8. YAP1 ablation by genetic knock out or ASO in GC tumor cells increased PDL-1 expression. Table S1. Primers used in this study. Table S2. Antibodies used in this study for Western blot, Immunoprecipitation, and Immunofluorescent staining. [file NIHMS2182360-supplement-Supplementary_Material.docx]

Original Research

**YAP1 Depletion Enhances TAZ and its Complexation with TEAD4 and AP-1 heterodimer C-JUN/FOSB in Gastric Cancer Progression and Metastasis**

Jingjing Wu ^1,2^, Dipti Athavale ^3,4^, Curt Balch ^3,4^, Junsong Zhao ^3,4^, Gengyi Zou ^1^, Yibo Fan ^1^, Yanting Zhang ^3,4,5^, Joseph Zhao ^6^, Mikel Ghelfi ^3,4^, Anthony Pompetti ^3,4^, Gennaro Calendo ^3,4^, Ailing Scott ^1^, Shan Shao ^1^, Xiaodan Yao ^3,4^, Melissa Pool Pizzi ^1^, Christopher Vellano ^7^, Vladimir Khazak ^8^, Sheng Zhang ^2^, Timothy A Yap ^9^, Shilpa S Dhar ^1^, Raghav Sundar ^6^, Francis Spitz ^4,5,10^, Generosa Grana ^4,10^, Jaffer A. Ajani ^1,*^, Shumei Song ^3,4,5,10,11,*^

1. Department of GI Medical Oncology, The University of Texas MD Anderson Cancer Center, Houston,
TX 77030, USA; Emails: 4468726@qq.com (J.W); GZou@mdanderson.org (G.Z.); yfan25@central.uh.edu (Y.F.); awscott@mdanderson.org (A.S.); SShao@mdanderson.org (S.S.); mppizzi@mdanderson.org (M.P.P.); SSDhar@mdanderson.org (S.S.D.)

2. Department of Pathology, The First Affiliated Hospital of Fujian Medical University, Fuzhou, Fujian 350005, China; Email: zhgshg@126.com (S.Z.)

3. Coriell Institute for Medical Research, 403 Haddon Ave, Camden, NJ, 08103, USA;
Emails: dathavale@coriell.org (D.A.); cbalch@coriell.org (C.B.); jzhao@coriell.org (J.Z.); yzhang@coriell.org (Y.Z.); mghelfi@coriell.org (M.G.); apompetti@coriell.org (A.P.);
gcalendo@coriell.org (G.C.); xyao@coriell.org (X.Y.)

4. Camden Cancer Research Center, 403 Haddon Ave, Camden, NJ, 08103, USA;
Emails: Spitz-Francis@cooperhealth.edu (F.S.); grana-generosa@cooperhealth.edu (G.G.)

5. Departments of Biomedical Sciences and Surgery, Cooper Medical School of Rowan University,
401 Broadway, Camden, NJ, 08103, USA

6. Gastroesophageal Cancer Program, Yale School of Medicine and Yale Cancer Center, PO Box 208028, New Haven, CT 06520-8028, USA; Emails: josephjzhao@u.nus.edu (J.Z.); raghav.sundar@yale.edu (R.S.)

7. Research Planning and Dev Traction, The University of Texas MD Anderson Cancer Center, Houston, TX 77030, USA; Email: CPVellano@mdanderson.org (C.V.)

8. NexusPharma Inc. 17 Black Forest Road, Hamilton, NJ 08691, USA;
Email: vladimir.khazak@gd3services.com (V.K.)

9. Department of Investigational Cancer (Phase 1 program), The University of Texas MD Anderson Cancer Center, Houston, TX 77030, USA; Email: TYap@mdanderson.org (T.A.P.)

10. MD Anderson Cancer Center at Cooper, Cooper University Hospital, 2 Cooper Plaza, Camden, NJ, 08103, USA

11. Department of Cancer Pharmacology, Rutgers Cancer Institute, 195 Little Albany St, New Brunswick, NJ 08901, USA

*** Correspondence:** Shumei Song; Email: [ssong@coriell.org](mailto:ssong@coriell.org); Jaffer A. Ajani; Email: jajani@mdanderson.org

**Supplementary Materials**

The following supplementary materials are available on the website of this paper:

1. Figure S1. YAP1 and TAZ are highly expressed and significantly correlated in GC tumor tissues and PDXs.

2. Figure S2. Co-expression of YAP1 and TAZ in representative GCPM samples.

3. Figure S3. High expression of TEAD1-TEAD4, the transcription factors (TFs) for YAP1/TAZ, in primary tumors and metastatic tissues.

4. Figure S4. Effects of YAP1 ASO on YAP1 targets and on tumor cell colony formation.

5. Figure S5. TAZ ASO specifically suppresses TAZ expression and inhibits GA0518 cell colony formation.

6. Figure S6. Cotreatment of GC tumor cells using YAP1 and TAZ ASOs effectively suppresses both YAP1 and TAZ expression and suppresses tumor cell growth in YAP1-high GC cells.

7. Figure S7. ASO co-targeting of YAP and TAZ significantly attenuates tumor growth in an additional PDX.

8. Figure S8. YAP1 ablation by genetic knockout or ASO in GC tumor cells increases PD-L1 expression.

9. Table S1. Primers used in this study.

10. Table S2. Antibodies used in this study for Western blot, immunoprecipitation, and immunofluorescent staining.

**
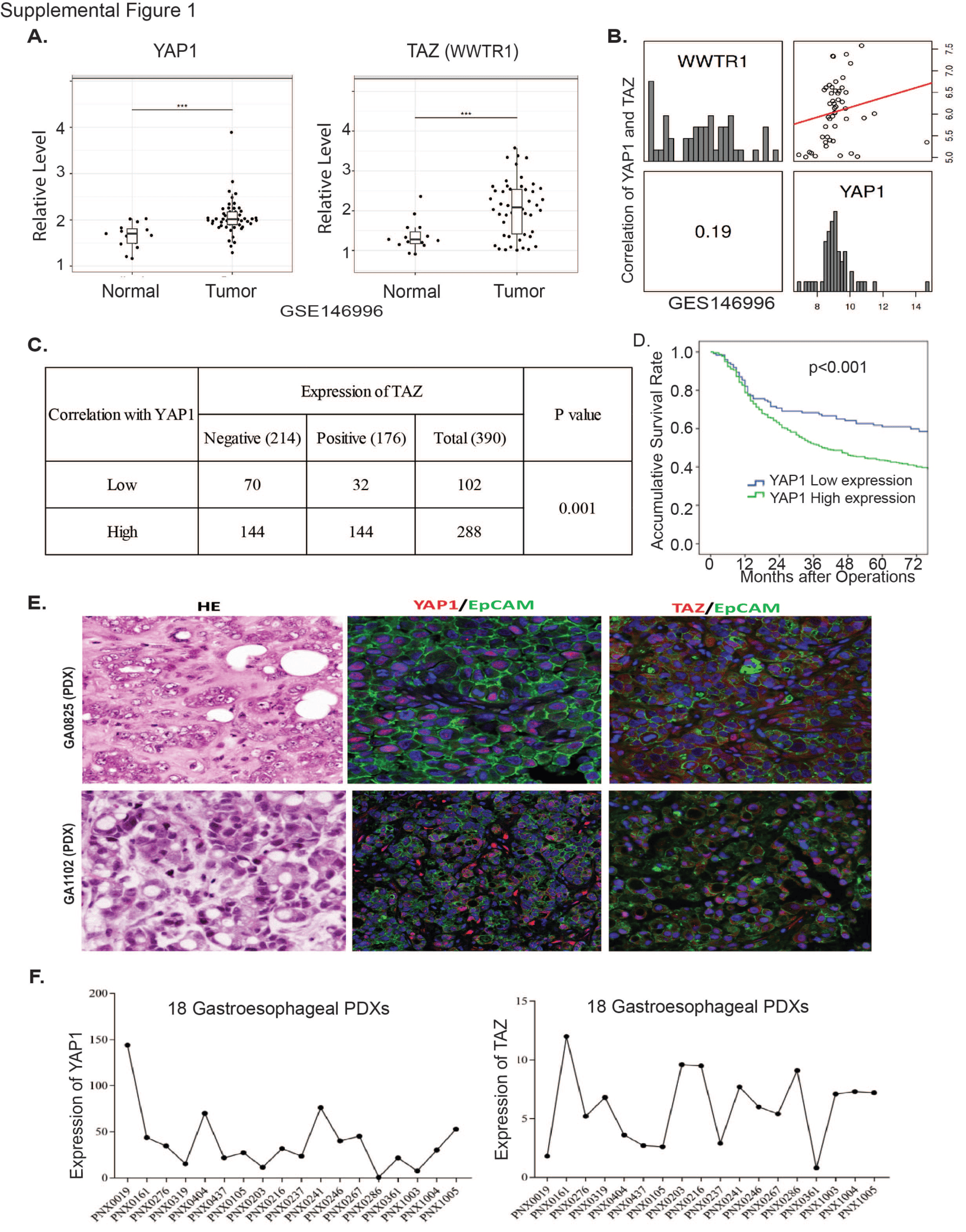
**

**Figure S1. YAP1 and TAZ are highly expressed and significantly correlated in GC tumor tissues and PDXs. (A)** Increased expression of YAP1 or TAZ (WWTR1) in GC tumor tissues compared to normal tissues from a public GC dataset (GSE146996). **(B)** Both YAP1 and TAZ are positively correlated in the same dataset. **(C)** Correlation of YAP1 and TAZ expression in our own TMA of 390 GC cases stained by IHC and statistically analyzed (p=0.001); **(D)** High expression of YAP1 is significantly associated with short survival (p<0.001) in the same GC cohort, **(E)** Co-expression of YAP1/EpCAM or TAZ/EpCAM was determined by co-IF in two representative PDXs; **(F)** mRNA expression of YAP1 or TAZ was determined in tumor tissues of 18 PDXs from gastroesophageal cancer patients.

**
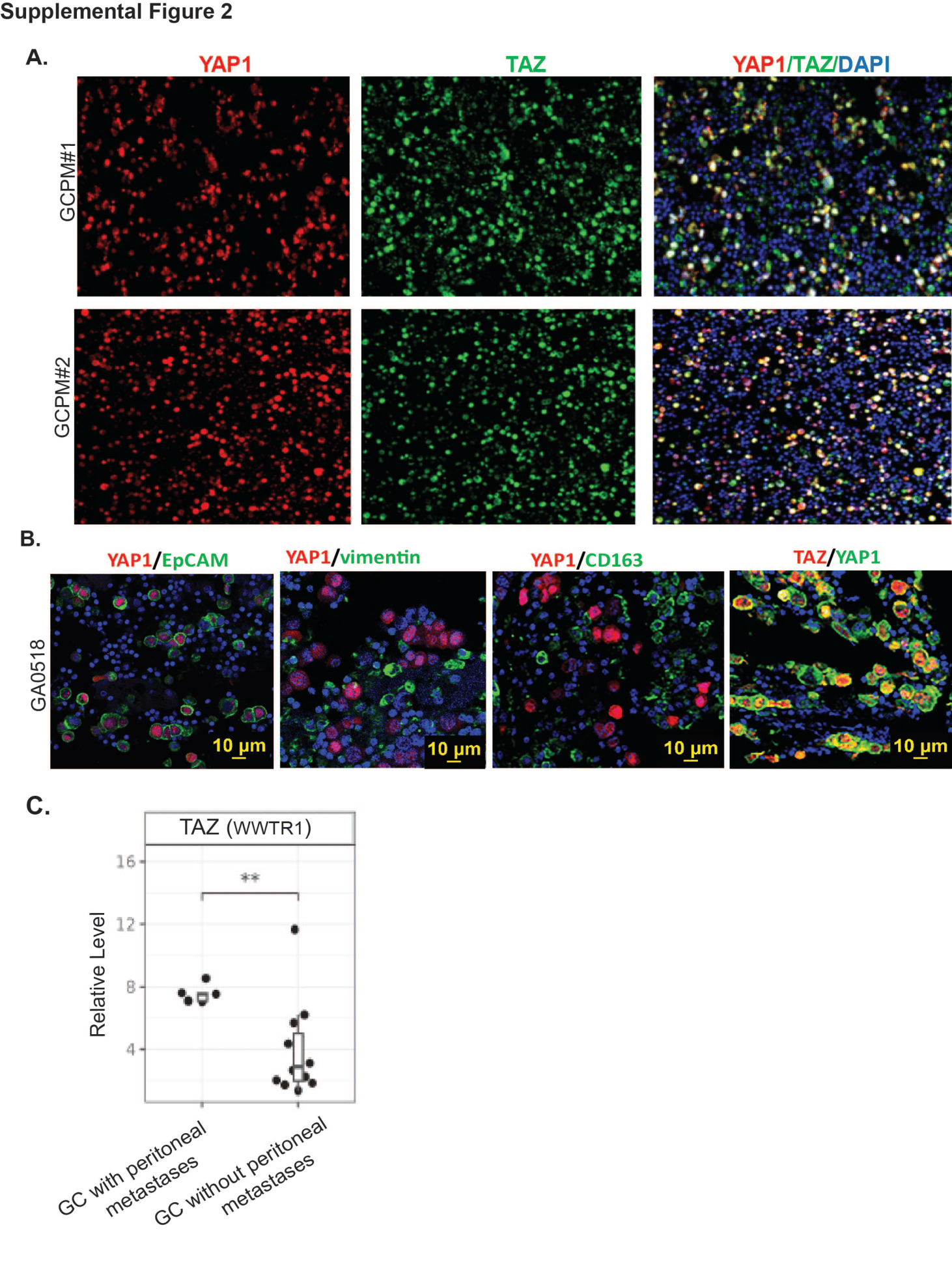
**

**Figure S2. Co-expression of YAP1 and TAZ in representative GCPM samples. (A)** Nuclear coexpression of YAP1 or TAZ in two representative GCPM specimens was determined by co-IF staining; **(B)** Coexpression of YAP1 with epithelial tumor marker EpCAM or TAZ or stromal marker vimentin and M2 macrophage marker CD163 in GA0518 GCPM cases; **(C)** TAZ expression was determined in GC with peritoneal metastases compared to GC without peritoneal metastases from a public GC dataset GSE289037.


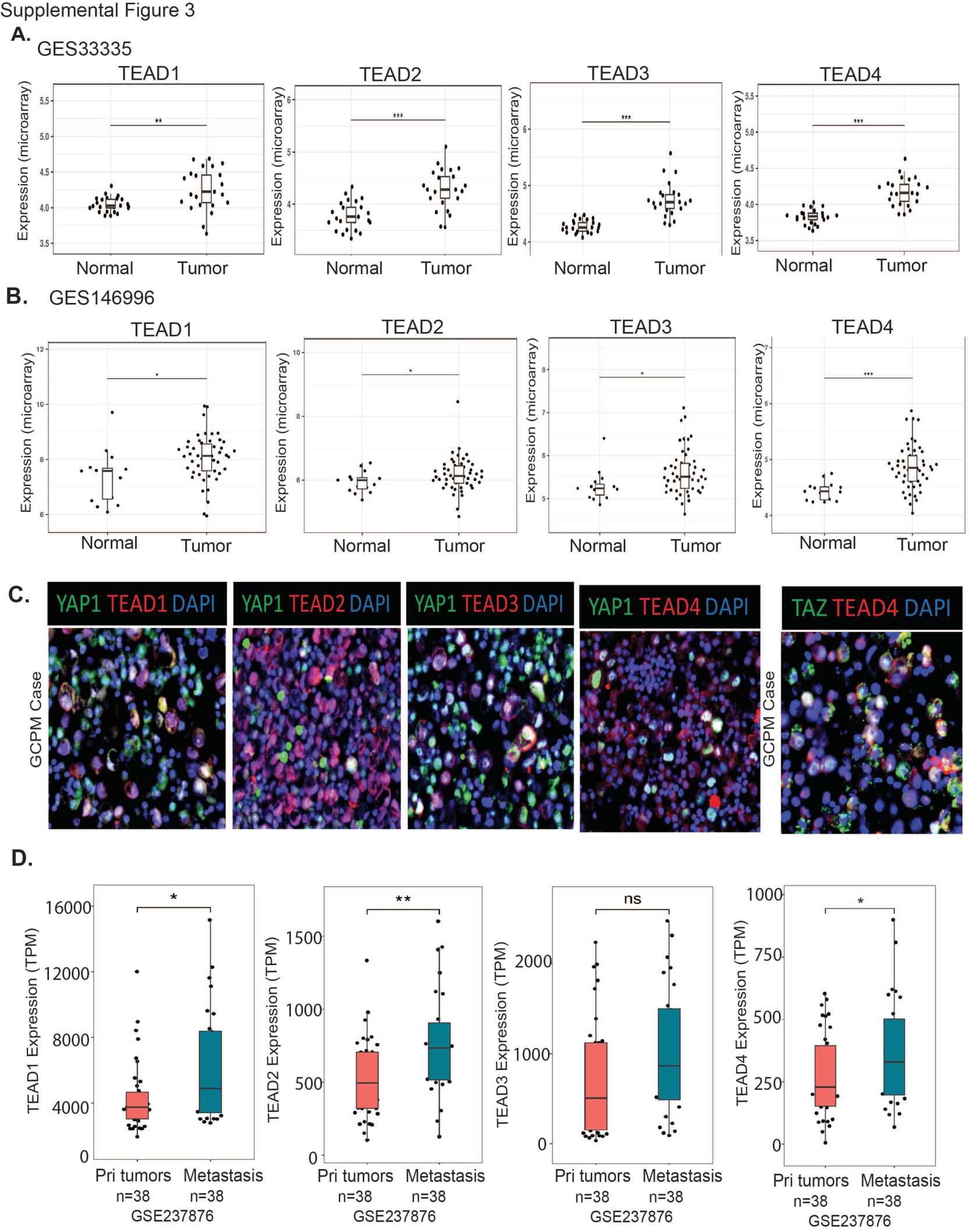


**Figure S3. High expression of TEAD1-TEAD4, transcription factors (TFs) for YAP1/TAZ, in primary tumors and metastatic tissues. (A)** Expression of TEAD1, TEAD2, TEAD3, and TEAD4 was significantly higher in GC tumor tissues compared to normal in the GES33335 GC cohort dataset. **(B)** Expression of TEAD1, TEAD2, TEAD3, and TEAD4 was significantly higher in GC tumor tissues compared to normal by analyzing the GC cohort GES146996 dataset; **(C)** Co-IF staining of YAP1 and TEAD1-TEAD4 or TAZ with TEAD4 in representative GCPM cases. **(D)** Expression of TEAD1-TEAD4 was analyzed in a GC cohort, GSE237876, by comparing primary tumors versus metastatic GC tumors. *p<0.05; **p<0.01.

**
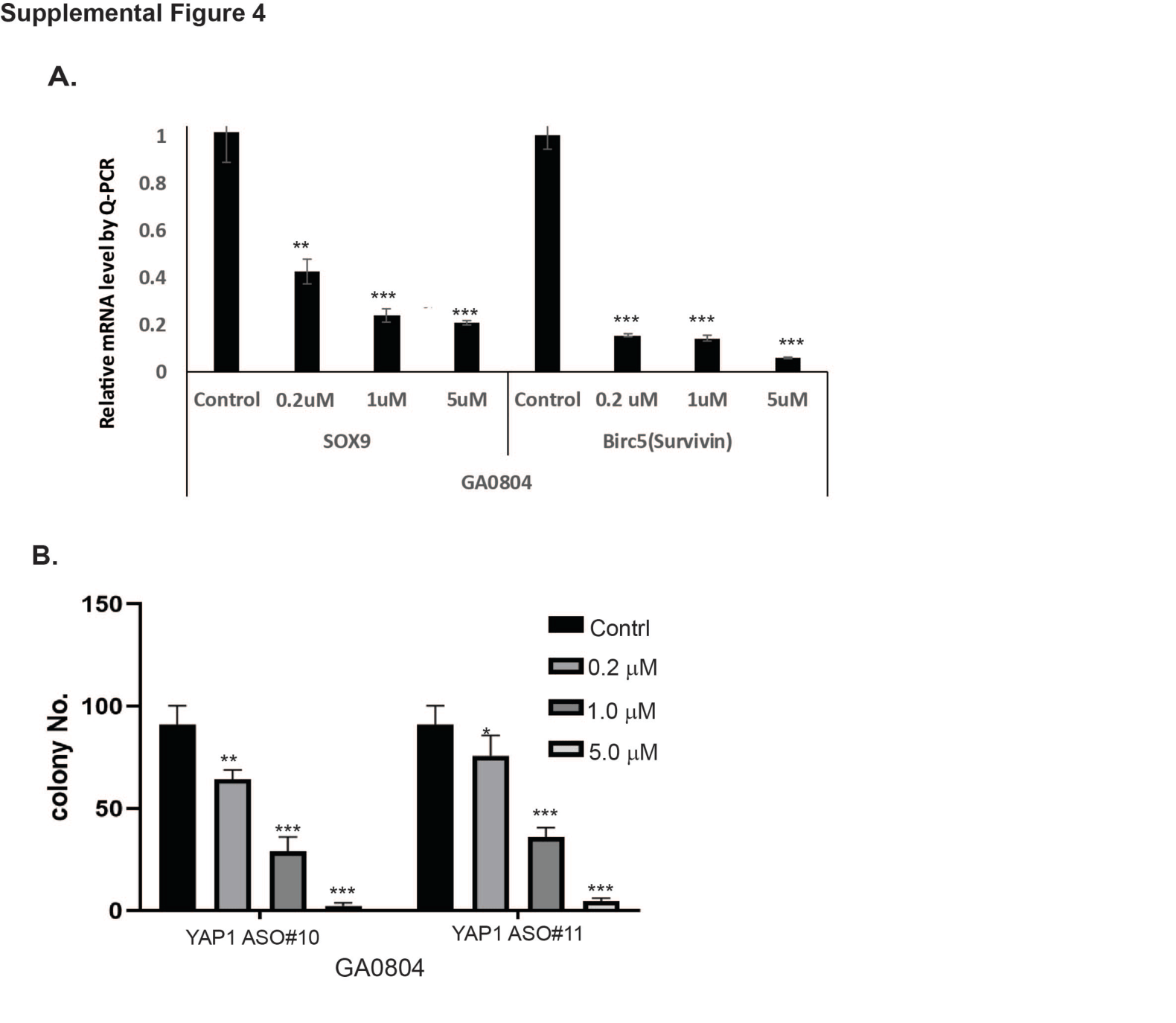
**

**Figure S4. Effects of YAP1 ASO on YAP1 targets and on tumor cell colony formation. (A)** mRNA levels of YAP1 targets, SOX9 and Birc5, were dose-dependently decreased by YAP1 ASO as determined by q-PCR in GA0804 tumor cells **p<0.01; ***p<0.001; **(B)** Colony formation was determined in GA0804 cells upon treatment with YAP1 ASO#10 or YAP1 ASO#11 at different dosages, as indicated. **p<0.01; ***p<0.001.


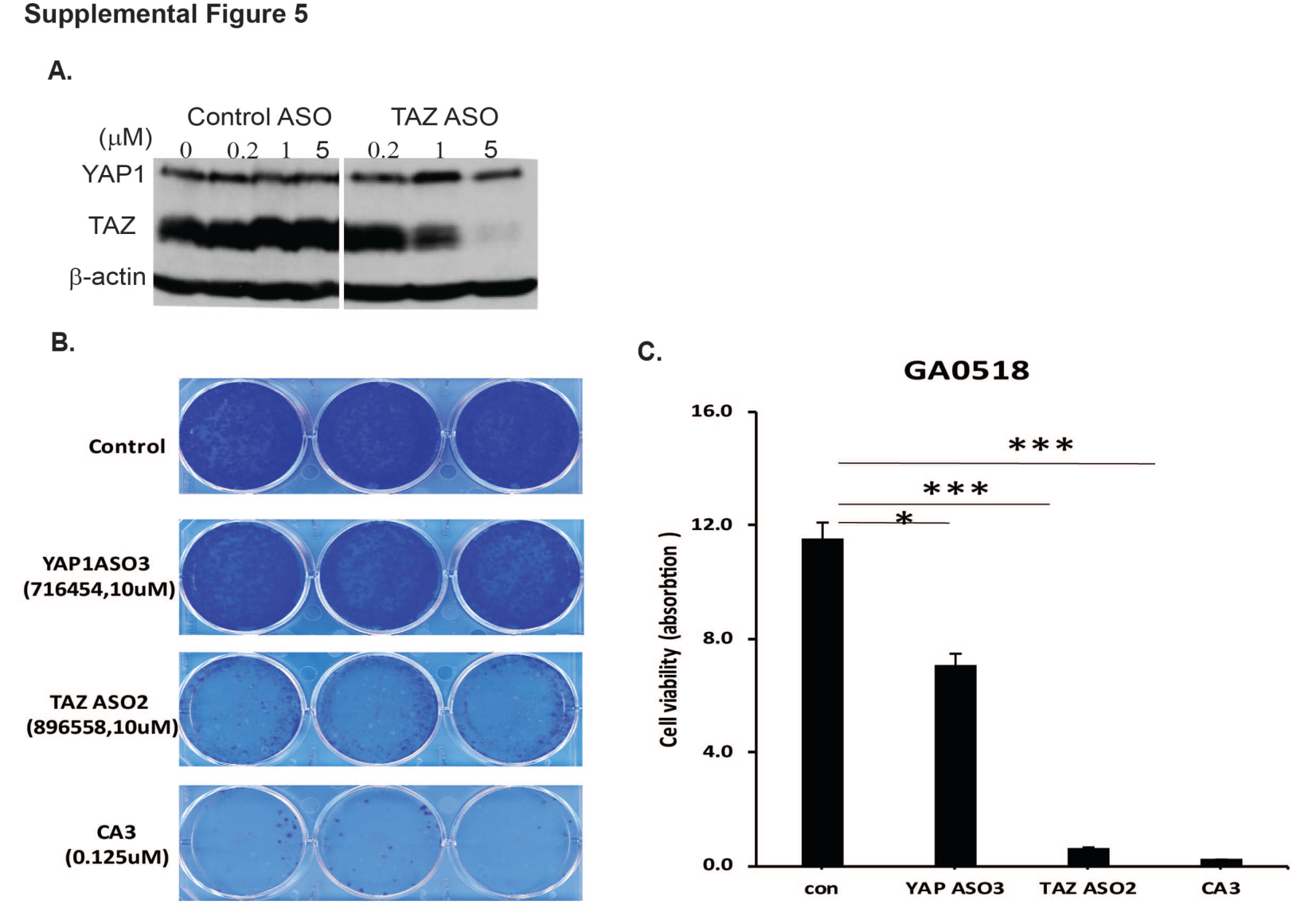


**Figure S5. TAZ ASO specifically suppresses TAZ expression and inhibits GA0518 cell colony formation. (A)** Expression of YAP1 and TAZ was determined in GA0518 cells by Western blot after treatment with TAZ ASO at the indicated dosages. **(B&C)** Demonstration of colony formation **(B)** and quantification **(C)** in GA0518 cells treated with YAP1 ASO, TAZ ASO, or a reported YAP1/TEAD inhibitor, CA3. *p<0.05, **p<0.01; ***p<0.001.


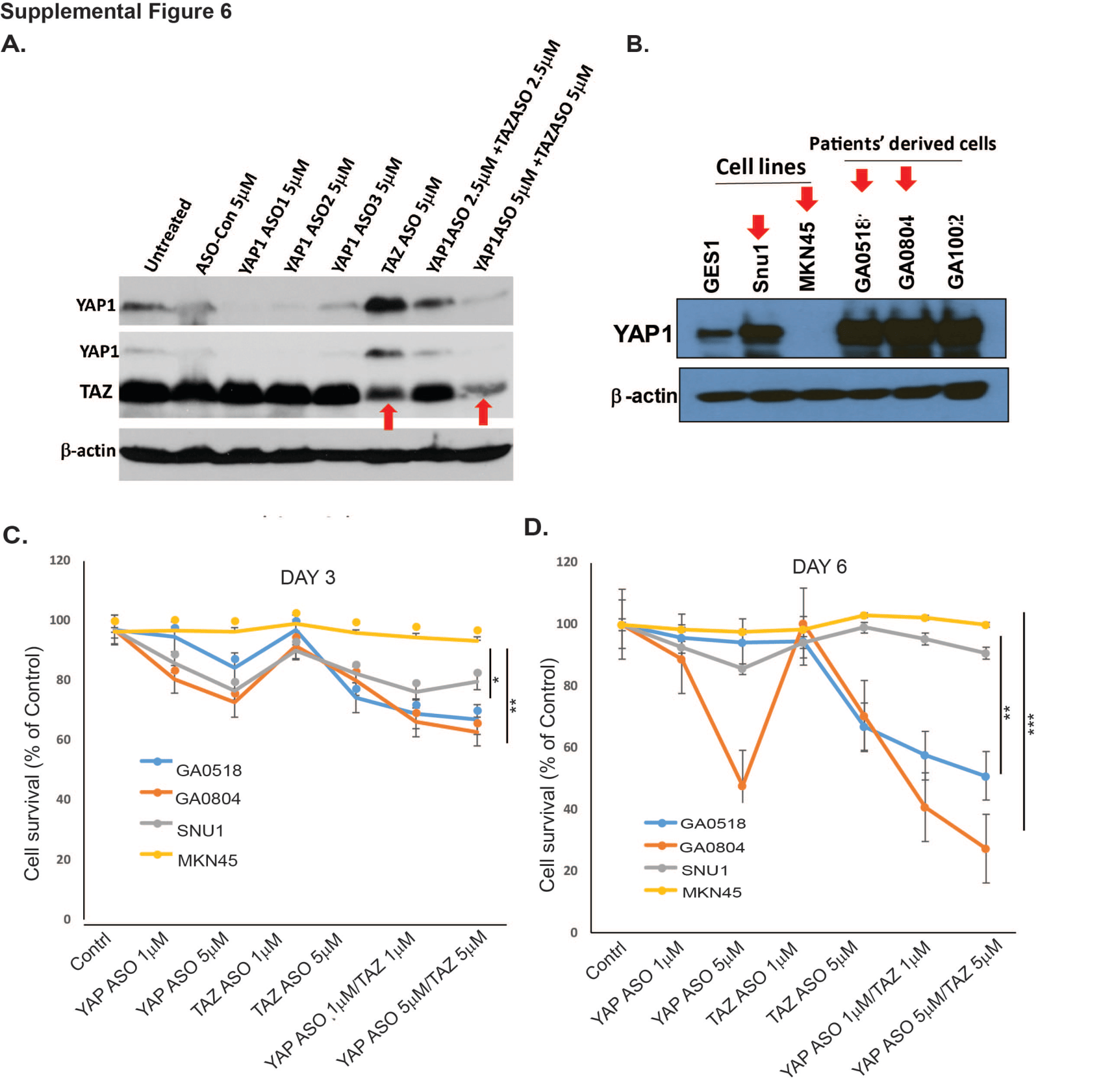


**Figure S6. Cotreatment of GC tumor cells with YAP1 and TAZ ASOs effectively suppresses both YAP1 and TAZ expression and suppresses tumor cell growth in YAP1-high GC cells. (A)** Expression of YAP1 and TAZ (red arrows) was determined in GA0518 cells by Western blot after treatment of three YAP1 ASOs, TAZ ASO, and their combination, at the indicated dosages; **(B)** Expression of YAP1 in GES1, two GC cell lines, and three GCPM-derived tumor cells was determined by Western blot; **(C&D)** Cell survival was detected by MTS assay in four GC cell lines with different YAP1 levels at the dosages indicated. *p<0.05; **p<0.01; ***p<0.001.


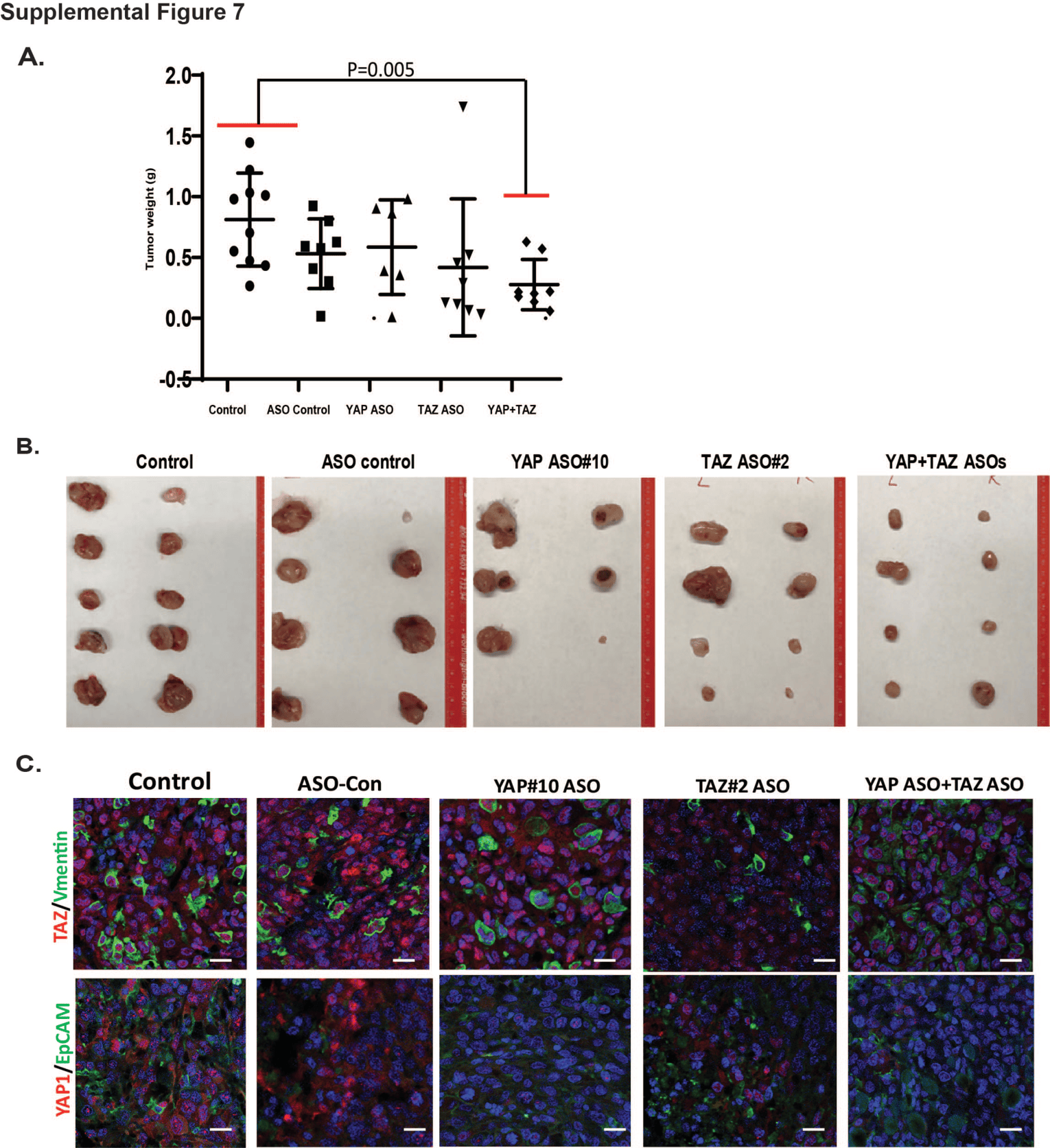


**Figure S7. ASO cotargeting of YAP and TAZ significantly attenuates tumor growth in an additional PDX.
(A)** Combined ASO inhibition of YAP1 and TAZ suppressed tumor weights in a GC PDX model; YAP1 ASO or TAZ ASO: 50mg/kg, the combination of YAP1 ASO (25mg/kg) and TAZ ASO (25mg/kg); 5 times a week for three weeks. **(B)** Tumor sizes in different treatment groups at the end of the experiment. C. Co-expression of TAZ/vimentin and YAP1/EpCAM as determined by IF staining in YAP1 ASO, TAZ ASO, or combination ASO-treated PDX tumors; Scale bar: 20 µm.

**
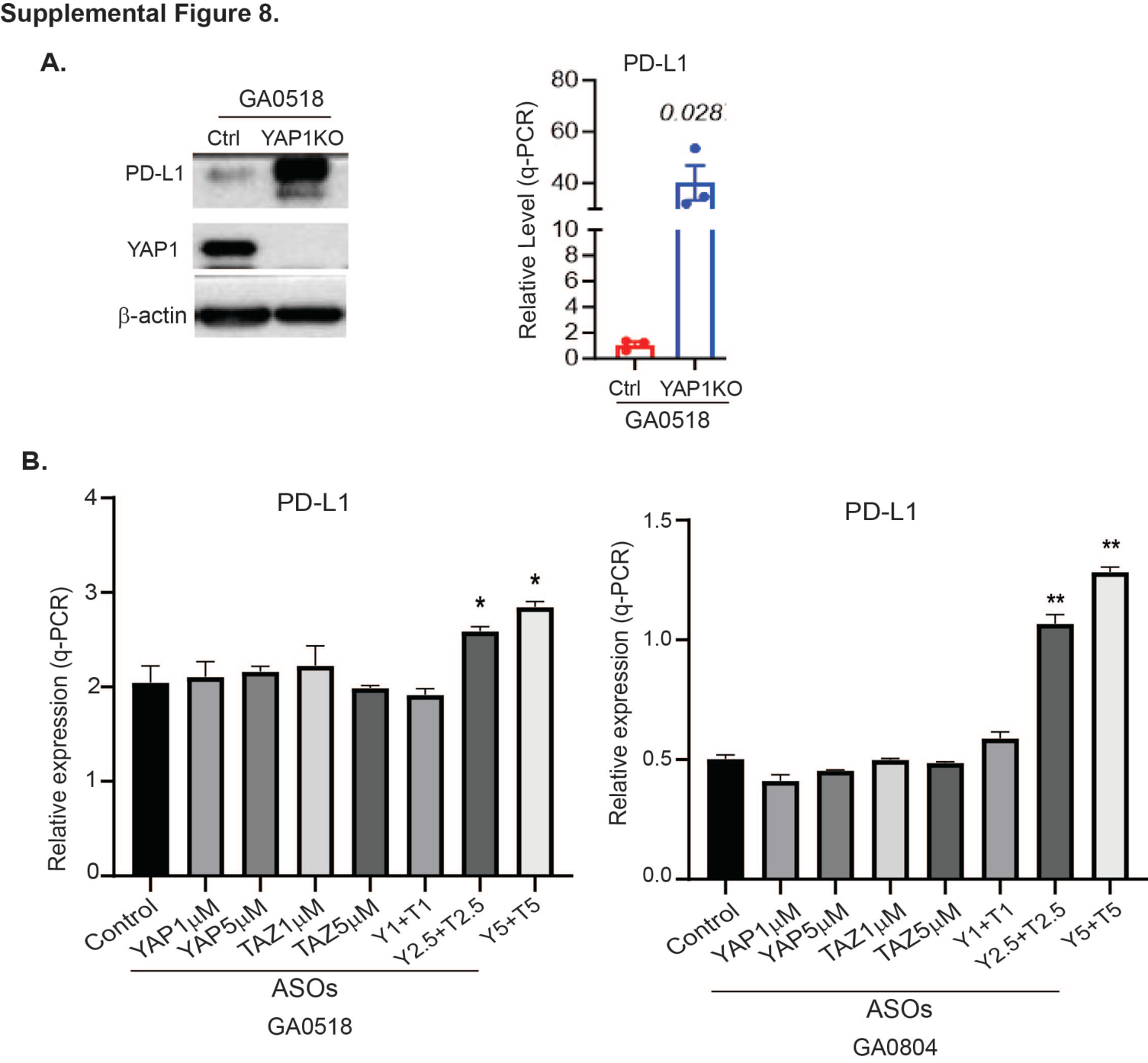
**

**Figure S8. YAP1 ablation by genetic knockout or ASO in GC tumor cells increased PD-L1 expression. (A)** Genetic depletion of YAP1 increased PD-L1 expression in GA0518 tumor cells by Western blot (left panel) and enhanced PD-L1 mRNA level by q-PCR (right panel); **(B)** Combined inhibition of YAP1 and TAZ using their ASOs increased PD-L1 expression in both GA0518 and GA0804 GCPM tumor cells by q-PCR. *P<0.05; **P<0.01.

**Table S1. Primers used in this study.**

| **Names of genes** | **5’ Forward** | **3’ Reverse** |
| --- | --- | --- |
| *Yap1* | 5’ ctgtcccagatgaacgtcac 3’ | 5’ ttctctggttcatggcaaaa 3’ |
| *WWTR1 (TAZ)* | 5’ TGGACCATGAGTACCAACCAC-3’ | 5’ GCGGGACTTGCCATATCTCAG3’ |
| *Cyr61* | 5’ AACGAGGACTGCAGCAAAA 3’ | 5’ TATTCACAGGGTCTGCCCTC 3’ |
| *CTGF* | 5’ GCTTGGCGATTTTAGGTGTC 3’ | 5’ CCACCCGAGTTACCAATGAC 3’ |
| *Birc5 (Survivin)* | 5’ GTTGCGCTTTCCTTTCTGTC 3’ | 5’ TCCGCAGTTTCCTCAAATTC 3’ |

**Table S2. Antibodies used in this study for Western blot, immunoprecipitation, and immunofluorescent staining.**

| **Antibody** | **Catalog No.** | **Company Name** |
| --- | --- | --- |
| YAP1 | #14074 | Cell Signaling |
| TAZ | NBP1-85067 | Novus |
| TEAD1 | #12292 | Cell Signaling |
| TEAD2 | SAB4503373 | Millipore Sigma |
| TEAD3 | ab75192 | Abcam |
| TEAD4 | ab58310 | Abcam |
| c-Jun | #9615 | Cell Signaling |
| FOSB | #2251 | Cell Signaling |
| Ki-67 | RM-9106-S1 | Thermo Scientific |
| CD8 | #98941 | Cell Signaling |
| CD163 | #25121 | Cell Signaling |
| CD206 | ab64693 | Abcam |
| Survivin | #2808 | Cell Signaling |
| SOX9 | AB5535 | Millipore Sigma |
| EpCAM | #2929 | Cell Signaling |
| Vimentin | sc-66002 | Santa Cruz Biotechnology |
| Snail | sc-271977 | Santa Cruz Biotechnology |
| β-Actin | MAB8929 | Biotechne, R&D Systems |
